# Is pulmonary embolism a chronic disease?

**DOI:** 10.1016/j.clinme.2025.100325

**Published:** 2025-05-09

**Authors:** Gerard Gurumurthy, Lianna Reynolds, Kerstin de Wit, Lara N. Roberts, Jecko Thachil

**Affiliations:** a University of Manchester, Manchester, UK; b Paediatric Haematology, Royal Manchester Children’s Hospital, Manchester, UK; c Department of Emergency Medicine, Queen’s University, Kingston, Ontario, Canada; d Division of Emergency Medicine, Department of Medicine, McMaster University, Hamilton, Canada; e King’s Thrombosis Centre, Department of Haematological Medicine, King’s College Hospital NHS, Foundation Trust, London, United Kingdom; f Institute of Pharmaceutical Sciences, King’s College London, London, United Kingdom; g MAHSC Professor, University of Manchester, Manchester, United Kingdom

**Keywords:** Pulmonary embolism, Post-pulmonary embolism syndrome, Chronic thromboembolic pulmonary hypertension (CTEPH), Chronic thromboembolic disease

## Abstract

Pulmonary embolism (PE) is often regarded as an acute disorder, yet emerging evidence underscores its chronic trajectory. Many survivors endure long-term complications, including recurrent thrombosis, persistent dyspnoea and psychosocial challenges. These sequelae impair functional capacity and quality of life long after the initial event. To address these issues, we suggest that clinicians should adopt an integrated, multidisciplinary model that includes risk stratification for recurrence, structured follow-up, exercise rehabilitation, and support for psychological challenges. Recognising the potential chronic sequalae of PE ultimately fosters comprehensive care aimed at reducing morbidity and improving long-term outcomes for survivors.

## Highlights

• Pulmonary embolism is often viewed as an acute event, yet growing evidence shows that it can have long-lasting physical and psychological consequences.
• Many survivors experience complications such as chronic thromboembolic pulmonary hypertension, persistent dyspnoea and recurrent thrombosis that significantly reduce their quality of life.
• Balancing the benefits of ongoing anticoagulation with the associated bleeding risks requires individualised risk assessment and regular follow-up.
• Targeted post-PE programmes, including supervised exercise and physiotherapy, can improve long-term function and help break the cycle of inactivity and fear of exertion.
• Psychological factors, including anxiety and depression, frequently arise after PE. Therefore, early identification and supportive interventions are key to comprehensive care.

## Graphical abstract

Pulmonary embolism is increasingly recognised as a condition with chronic implications. Survivors often face a spectrum of long-term complications, including recurrent thrombotic events, persistent dyspnoea, psychological distress, and chronic conditions such as chronic thromboembolic pulmonary hypertension (CTEPH) and chronic thromboembolic disease (CTED). Effective long-term management requires a multidisciplinary approach encompassing risk stratification, structured follow-up, rehabilitation programmes and psychosocial support.

**Figure:**
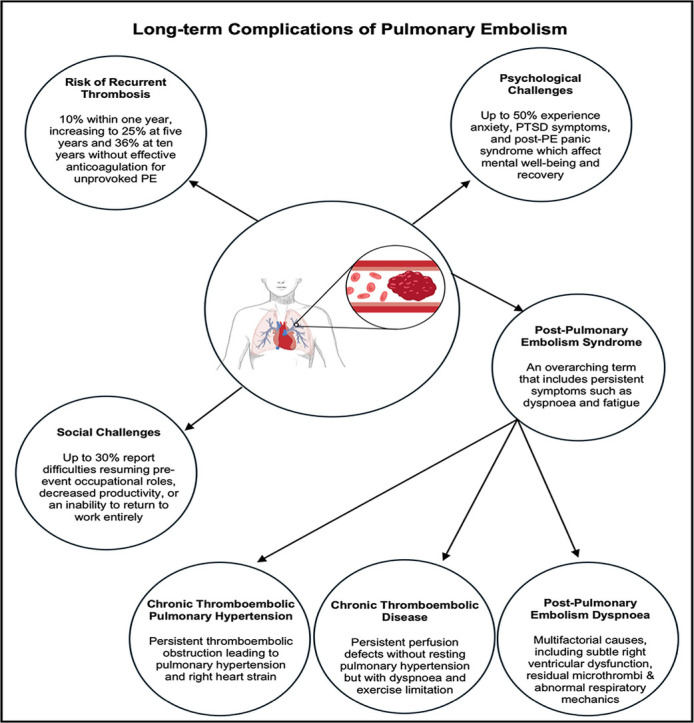

## Introduction

Pulmonary embolism (PE) is an acute, potentially life-threatening condition caused by obstruction of the pulmonary arteries, most commonly by thrombi originating in the deep veins of the lower extremities.^1^^,^^2^ It is the third most common cardiovascular disorder (second after myocardial infarction and stroke) with an incidence of one to two cases per 1,000 individuals annually.^1^, ^2^, ^3^ The mainstay of acute PE management includes prompt anticoagulation. Patients with massive PE and evidence of right ventricular compromise may require systemic thrombolysis or, in select cases, catheter-directed reperfusion therapy or surgical embolectomy.^2^^,^^4^^,^^5^ Advances in diagnostic modalities, including computed tomography pulmonary angiography (CTPA), and the advent of direct oral anticoagulants (DOACs) have significantly improved early recognition and short-term outcomes respectively.^2^^,^^4^, ^5^, ^6^

A growing body of evidence demonstrates that PE frequently leads to persistent complications that extend beyond the acute phase, suggesting that it is not merely a transient event. Instead, many survivors experience a chronic trajectory marked by recurrent thrombotic events, incomplete resolution of symptoms, reduced functional capacity, compromised quality of life, and psychological distress. This is collectively termed post-pulmonary embolism syndrome’ (PPES).^7^, ^8^, ^9^ Three conditions broadly encompass this spectrum: chronic thromboembolic pulmonary hypertension (CTEPH), chronic thromboembolic disease (CTED) and post-PE dyspnoea.^9^, ^10^, ^11^, ^12^ CTEPH arises when unresolved thrombi become fibrotic, permanently obstructing or narrowing pulmonary vessels and causing elevated pulmonary arterial pressures and right ventricular strain.^10^, ^11^, ^12^ In contrast, CTED involves reduced exercise tolerance and exertional dyspnoea without meeting resting haemodynamic criteria for pulmonary hypertension.^10^, ^11^, ^12^ Lastly, post-PE-related dyspnoea encompasses the above-mentioned deconditioning which patients may face without any evidence of pulmonary vascular disease.^7^ These chronic manifestations underscore that PE is not merely a one-time event, but a condition with potentially ongoing morbidity.

Re-envisioning PE as a chronic disorder has vital implications for clinical practice, shifting emphasis from a single, acute intervention towards integrated, long-term management strategies. This review aims to provide a comprehensive overview of PE as a condition with chronic implications. The scope includes a critical examination of the long-term physical and psychosocial impact on patients’ lives. We also elucidate pathways for healthcare providers to identify and manage patients with PPES. By exploring these dimensions, the article encourages clinicians and researchers to adopt a more holistic and sustained approach to PE management, ultimately improving long-term outcomes and quality of life for survivors.

## Likelihood of thrombosis recurrence

### Risk of recurrence

Despite effective acute treatment, the long-term risk of venous thromboembolism recurrence remains considerable, therefore secondary prophylaxis is considered. Without secondary prophylaxis, patients with unprovoked PE may face recurrence rates as high as 10% within 1 year, increasing to 25% at 5 years and 36% at 10 years.^13^ Persistent or uncorrected risk factors, including malignancy, antiphospholipid syndrome, raised body mass index (BMI) and chronic inflammatory disorders, further elevate these rates.^14^, ^15^, ^16^ For patients with strong provoking factors, such as recent surgery or trauma, anticoagulation may be safely discontinued after 3 months, as the recurrence risk is lower in this group. Anticoagulation duration should otherwise match individual risk profiles to balance the benefits of preventing recurrence with the risks of bleeding second to anticoagulation.^14^ Periodic reassessment is warranted due to the dynamic nature of these risk factors.^4^^,^^17^

### Risk stratification tools for unprovoked PE

The management of unprovoked PE requires careful balancing between the prevention of recurrent venous thromboembolism (VTE) and the risk of bleeding. Both outcomes carry significant morbidity and mortality, necessitating a nuanced approach to anticoagulation therapy. While the risk of recurrence has traditionally been the primary focus in guiding these decisions, the potential for bleeding complications requires equal attention. This dual consideration underscores the importance of integrating bleeding risk assessments into clinical decision making.

The risk of major bleeding is highest within the first 3 months of treatment, with an estimated occurrence rate of 2% and a fatality rate of around 10%.^18^, ^19^, ^20^ The International Society of Thrombosis and Haemostasis therefore recommends that an initial assessment of bleeding risk be conducted at the time of VTE diagnosis and prior to initiating anticoagulant therapy for newly diagnosed cases.^21^ Modifiable factors include hypertension, use of platelet inhibitors or non-steroidal anti-inflammatory drugs, anaemia and renal insufficiency, all of which present opportunities for intervention to reduce bleeding risk. Non-modifiable factors such as prior bleeding, advanced age, sex and prior stroke also significantly influence risk, but require careful management of anticoagulant therapy to minimise harm.^21^^,^^22^ Risk prediction tools such as VTE-BLEED,^23^ RIETE^24^ and HAS-BLED^25^ have been developed and externally validated to evaluate bleeding risk in patients undergoing anticoagulation. These models incorporate key predictors like age, renal function, prior bleeding, cancer and hypertension, offering a structured framework for risk assessment.

Ultimately, balancing the risk of PE recurrence against bleeding complications requires an individualised approach to VTE care by encouraging periodic re-evaluation of both recurrence and bleeding risks. In doing so, anticoagulation decisions transform from a one-time judgment into a proactive, long-term management strategy. This iterative process ensures that patients benefit from tailored therapy that maximises efficacy while minimising harm. Incorporating bleeding risk assessments into routine practice not only refines patient selection for extended anticoagulation, but also enhances the safety and precision of VTE management overall.

## Post-pulmonary embolism dyspnoea

Persistent dyspnoea is common and can affect up to 50% of PE survivors, even without overt vascular obstructions or established CTEPH/CTED.^7^^,^^26^, ^27^, ^28^ The underlying mechanisms are often multifactorial, including subtle right or left ventricular dysfunction, residual microthrombi, abnormal respiratory mechanics and deconditioning.^9^^,^^29^, ^30^, ^31^ Over time, these factors contribute to a cycle of reduced activity, progressive loss of cardiopulmonary fitness, and ongoing symptom burden. Addressing persistent dyspnoea as a chronic challenge emphasises the need for continuous monitoring and interventions that extend well beyond initial hospital discharge.

**Fig. 1:**
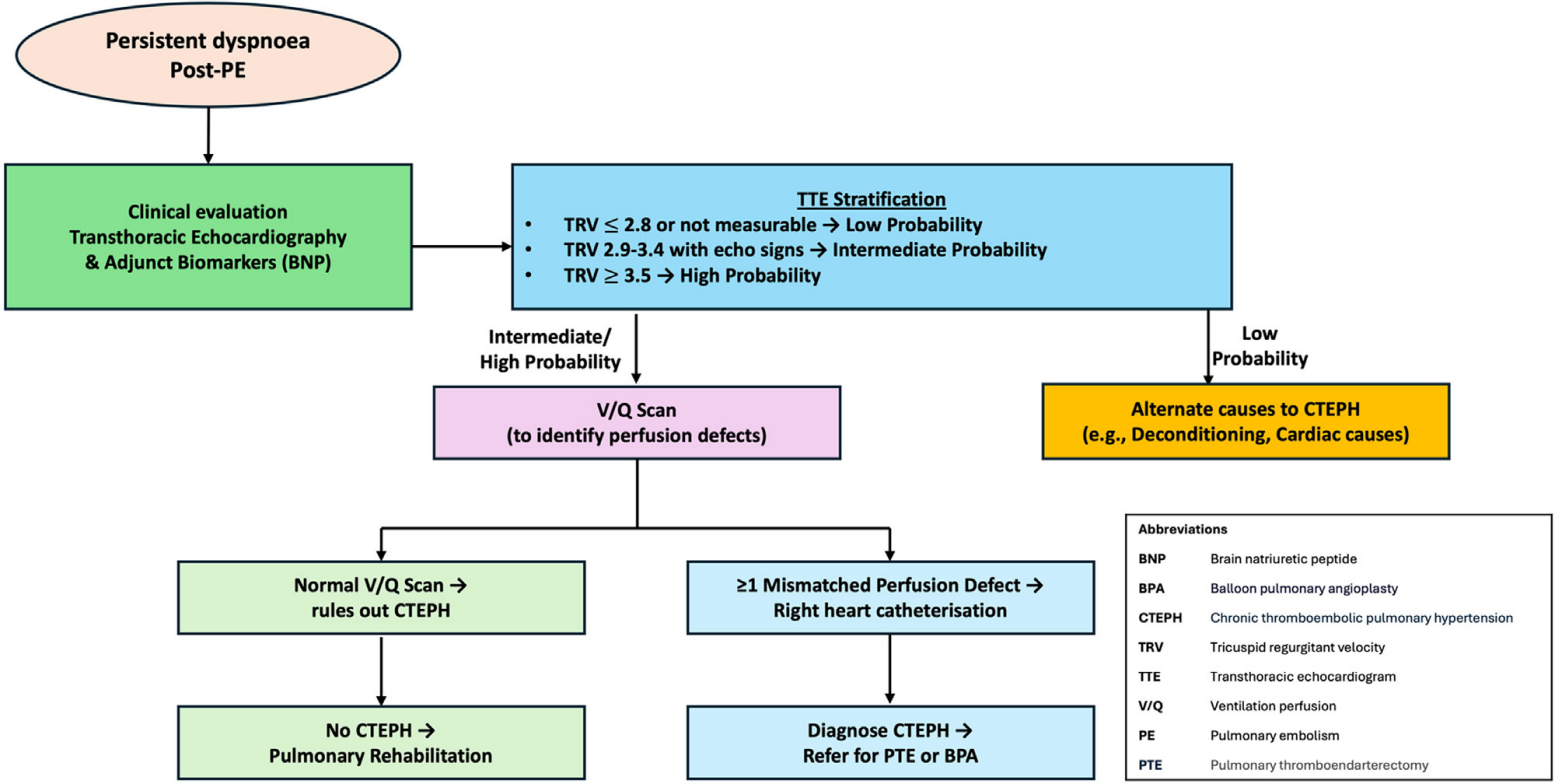
Algorithm for diagnosing and managing persistent dyspnoea post-pulmonary embolism.

As outlined in Fig. 1, investigating post-PE dyspnoea begins with ruling out CTEPH as the primary cause of persistent symptoms. Initial diagnostic steps typically involve transthoracic echocardiography to evaluate right ventricular function and estimate pulmonary artery pressures. Patients with suggestive findings may require further assessment with advanced imaging or invasive testing as detailed later on.

Supervised rehabilitation programmes, combining aerobic and resistance exercises, have been suggested to demonstrate improvements in VO_2_ max, exercise tolerance and quality of life.^32^, ^33^, ^34^ There have been a number of feasibility studies and safety studies which have identified that physiotherapy and nurse specialist intervention are safe and feasible.^27^^,^^33^ A single-arm trial identified health improvements following pulmonary rehabilitation for patients with persisting symptoms following PE.^33^ Control trials show mixed results, likely due to sample size. One study showed no difference between an 8-week rehabilitation intervention compared to a consultation with a nurse specialist.^35^ Another suggests benefits including increased 6-minute walk distance and enhanced inspiratory muscle strength.^32^ Furthermore, the long-term sustainability and effectiveness of these interventions have not been studied. These remain areas requiring further investigation. It is argued that these structured interventions could help break the cycle of inactivity and fear-driven avoidance of exertion, gradually restoring confidence in physical capabilities. Regular follow-up, exercise progression and ongoing support from a multidisciplinary team (including physiotherapists, pulmonary rehabilitation specialists and psychologists) exemplify how addressing post-PE dyspnoea fits neatly into a chronic care model.

## Chronic thromboembolic pulmonary hypertension

Chronic thromboembolic pulmonary hypertension (CTEPH) exemplifies how PE can evolve into a fixed, chronic vascular pathology. It affects approximately 1–5% of survivors.^10^ CTEPH arises when unresolved thrombi become fibrosed, leading to permanent pulmonary vascular obstruction, elevated pulmonary vascular resistance and remodelling, and progressive right ventricular dysfunction.^27^^,^^36^^,^^37^ The natural history of CTEPH is characterised by persistent, and often worsening, haemodynamic impairment. Without intervention, the condition carries up to a 30% 2-year mortality.^10^^,^^38^^,^^39^

Transthoracic echocardiography is the main initial investigation, recommended at 3 months or later if new or progressive symptoms arise, to assess the probability of pulmonary hypertension. Key echocardiographic parameters include the tricuspid regurgitation velocity (TRV) and pulmonary artery systolic pressure (PASP). It is suggested that TR is linked to poorer prognosis, reduced RV function, and lower RV-pulmonary artery coupling even when tricuspid annular plane systolic excursion (TAPSE) remains preserved. Consequently, persistently significant TR should prompt more aggressive therapy for their pulmonary hypertension, irrespective of standard four-strata risk scores.^40^ According to the 2022 ESC/ERS guidelines, a TRV >2.8 m/s or other supportive echocardiographic signs (eg right ventricular dilation or dysfunction) should trigger further evaluation for pulmonary hypertension.^41^ Additionally, biomarkers such as B-type natriuretic peptide (BNP) can be valuable adjuncts in detecting right ventricular strain and assessing disease severity, with elevated levels supporting the need for expedited investigation. Patients with high suspicion of pulmonary hypertension require expedited referral to a specialist pulmonary hypertension service for further investigation, including ventilation-perfusion (V/Q) scanning, right heart catheterisation, and detailed imaging of the pulmonary vasculature.^27^^,^^36^^,^^38^^,^^39^

Pulmonary thromboendarterectomy (PTE) offers potential cure or significant improvement for eligible CTEPH patients.^27^^,^^36^^,^^38^ For inoperable cases or those with residual disease after surgery, balloon pulmonary angioplasty (BPA) and targeted medical therapies (e, riociguat) provide alternative long-term management strategies.^27^^,^^37^^,^^38^^,^^42^ These therapies do not resolve all the problems; they often necessitate ongoing follow-up, optimisation of treatment regimens, and rehabilitation to maintain functional gains.^43^

## Chronic thromboembolic disease

CTED refers to persistent perfusion or vascular abnormalities without meeting the haemodynamic criteria for CTEPH. It is suggested that this subtle but persistent form of vascular impairment can limit activities, reduce work capacity, and detract from overall wellbeing indefinitely. Patients with CTED may experience exertional dyspnoea and reduced functional capacity without meeting haemodynamic criteria for pulmonary hypertension at rest.^10^, ^11^, ^12^^,^^27^ However, no consistent relationship between residual thromboembolic obstruction on imaging and functional limitation at 6–12 months post-PE has been identified.^44^^,^^45^ This suggests that persistent abnormalities observed on imaging do not always translate into exercise intolerance or reduced quality of life. It is also important to note that CTED can be identified on imaging in patients with no overt symptoms.^44^^,^^45^ The chronic sequelae of PE are not confined to the most severe manifestations like CTEPH and are likely more nuanced than these proposed mechanisms and findings.

Diagnosing CTED relies on advanced testing, including cardiopulmonary exercise testing (CPET) or stress echocardiography to reveal exercise-induced pulmonary hypertension or V/Q mismatches.^27^^,^^35^^,^^46^^,^^47^ Continuous clinical monitoring and reassessment are essential to detect subtle changes in exercise tolerance, pulmonary function and haemodynamic status post-PE. This ensures that management strategies remain appropriately tailored to the patient’s condition.

### Psychological impact after pulmonary embolism

#### Prevalence of psychological morbidity and chronic stressors

Psychological morbidity among PE survivors is increasingly recognised as a common problem. Anxiety, depression and post-traumatic stress disorder symptoms may arise due to the life-threatening nature of PE, lingering uncertainties about recurrence risk, and the acute physical limitations.^26^^,^^48^^,^^49^ Up to 50% of survivors may experience anxiety or depression, and 3–5% develop PTSD symptoms.^8^^,^^14^^,^^26^^,^^48^^,^^49^ Over time, these psychological stressors can compound, leading to chronic emotional distress that influences adherence to medical therapy, willingness to engage in rehabilitation, and overall quality of life.^50^

#### Post-PE psychology

Qualitative studies have highlighted how healthcare provider communication during the diagnosis and acute management of PE significantly shapes patients’ psychological responses. Many patients recall being told they had a ‘life-threatening clot’ or were ‘lucky to be alive’.^50^, ^51^, ^52^ The statement often instilled a lasting sense of fear and vulnerability. The urgent nature of PE diagnosis can heighten anxiety, especially if communicated with alarmist language or excessive medical jargon.^50^^,^^51^ Similarly, incomplete explanations or patient understanding about the diagnosis, prognosis or purpose of anticoagulation therapy left patients uncertain about their long-term risk of recurrence and the effectiveness of their treatment.^51^ Furthermore, a lack of structured follow-up or opportunities to ask questions about their condition contributed to lingering uncertainty and heightened psychological distress.^50^^,^^51^ Conversely, clear, empathetic communication (explaining the condition in accessible terms, outlining treatment plans and addressing immediate concerns) can reduce anxiety and foster emotional resilience. Effective healthcare provider communication at the time of diagnosis is, therefore, a critical yet often-overlooked factor in preventing long-term psychological morbidity among PE survivors.^51^ Patients report the persistent fear of recurrence, which exemplifies the intersection of physiological risk and psychological burden.^8^^,^^14^^,^^26^^,^^48^^,^^49^

Addressing post-PE psychological distress requires sustained patient education about recurrence risk, reassurance regarding the protective effects of anticoagulation, and the provision of coping strategies. Cognitive behavioural interventions, peer support groups and structured follow-up visits have been shown to help patients manage their fears and regain a sense of control. Importantly, healthcare providers must be equipped with the skills to deliver clear, empathetic and balanced communication at the time of diagnosis. This includes explaining the condition in accessible terms, providing context about prognosis, and creating space for patients to express their concerns and ask questions.

#### Screening, psychological interventions, and long-term support

The International Consortium for Health Outcomes Measurement (ICHOM) recommends the Pulmonary Embolism Quality of Life (PEmb-QoL),^8^ Patient-Reported Outcomes Measurement Information System (PROMIS) Scale v1.2 – Global Health,^53^ VEINES-QOL questionnaires^54^ and, if required, the PHQ-9^55^ and GAD-7 questionnaires^56^ as validated instruments to screen for the psychosocial impacts of post-PE.^57^ Once identified, cognitive behavioural therapy (CBT), mindfulness-based interventions, peer support groups, and patient education about the low likelihood of recurrence can reduce distress. Longer-term mental health follow-up, as part of a multidisciplinary team, ensures that psychological needs are addressed on a continuous basis.

### Impact of pulmonary embolism on daily life, work and social participation

#### Return to work and long-term functional integration

The persistent physical and psychological challenges faced by PE survivors can hinder reintegration into the workforce.^48^^,^^58^ Approximately 30% report difficulties resuming pre-event occupational roles, decreased productivity or an inability to return to work entirely.^7^^,^^26^^,^^59^ Over the long term, chronic dyspnoea, fatigue and fear of exertion may limit career progression and economic stability.^60^ A comprehensive assessment of the post-PE functionality with the ICHOM’s recommendations^57^ may be warranted in such instances. Once assessed, vocational rehabilitation programmes, workplace accommodations (eg flexible hours, reduced physical demands), and ongoing medical supervision can help mitigate these challenges, facilitating a gradual return to normal occupational functioning. Such adjustments mirror strategies used in other chronic conditions where sustained support systems are required for successful long-term re-engagement in work and society.^28^^,^^59^, ^60^, ^61^

#### Daily activities, social participation and sexual health

Beyond work, persistent symptoms and psychological distress may influence survivors’ engagement in hobbies, social events and family activities. Fear of symptom exacerbation, recurrence anxiety and reduced fitness can lead to withdrawal from previously enjoyed pursuits.^28^^,^^61^^,^^62^ Over time, this can erode social relationships, reduce life satisfaction and perpetuate isolation. Similarly, sexual health may be compromised, as individuals worry that physical exertion could trigger symptoms.^49^^,^^63^ Providing clear guidance on safe activities, incremental exercise progression, and assurance about the protective role of appropriate anticoagulation can empower patients to participate fully in their home and social lives. These ongoing lifestyle adaptations reinforce the notion that managing PE’s aftermath is a chronic, multidisciplinary endeavour, rooted in continuous patient engagement, education and psychosocial support.

## Investigation and management in post-pulmonary embolism

Persistent signs and symptoms after an acute PE, such as ongoing dyspnoea, exercise intolerance, chest discomfort and palpitations, frequently warrant targeted investigation and structured long-term care.^7^^,^^9^^,^^26^ These manifestations often indicate the possibility of PPES.^7^^,^^10^^,^^11^ Psychological sequelae can also trigger recurrent healthcare visits, underscoring the need for a holistic approach that recognises the interplay between physiological and psychological burdens.^26^^,^^48^^,^^49^

**Fig. 2:**
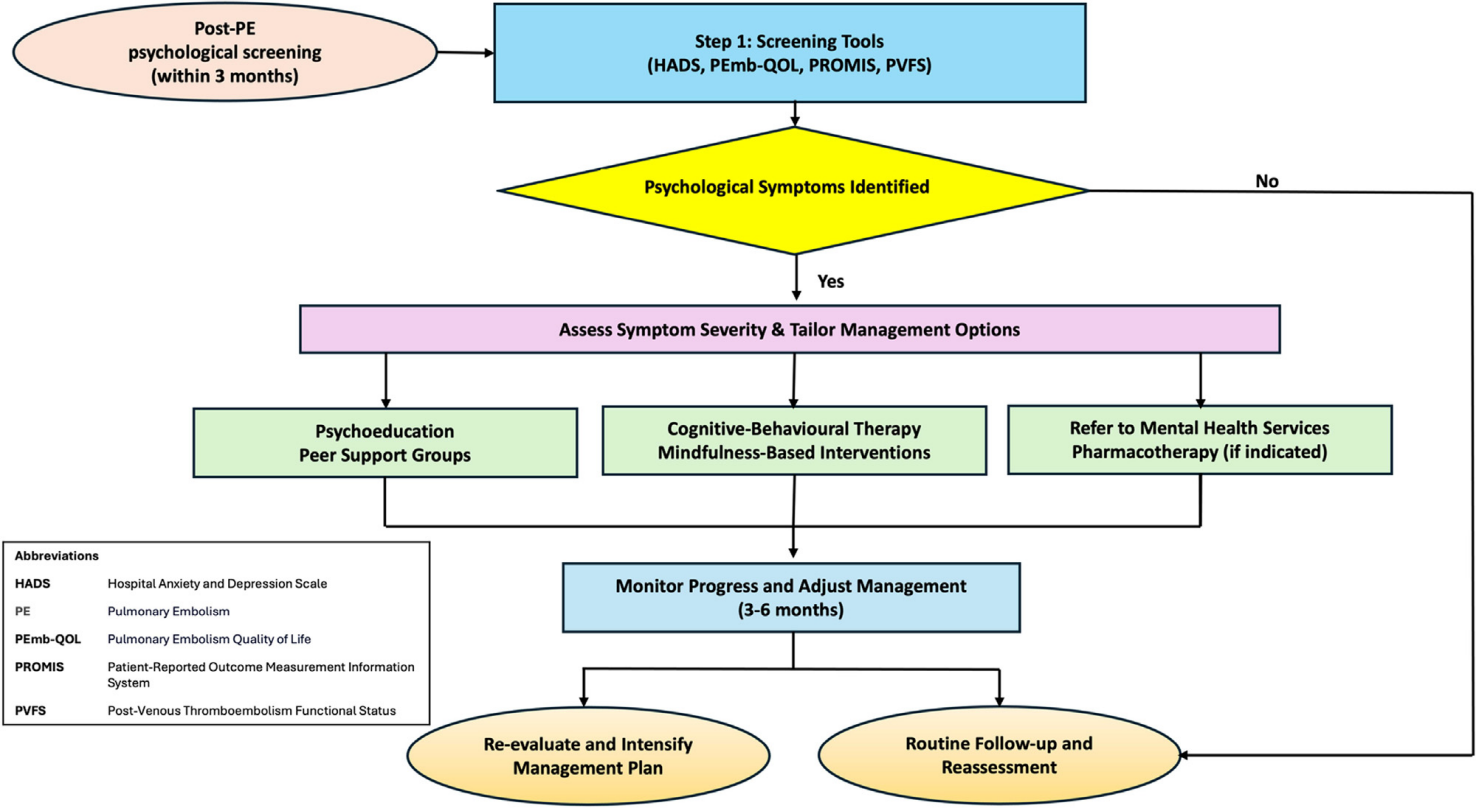
Psychological screening and management pathway post-pulmonary embolism.

It is ideal not to evaluate patients for PPES earlier than 3 months of anticoagulation.^7^^,^^9^^,^^36^ In the immediate weeks post-event, residual cardiopulmonary strain, incomplete thrombus resolution and deconditioning can mimic or mask chronic complications, leading to overestimation of true PPES if investigations occur prematurely.^64^ By waiting 3–6 months, clinicians can more accurately identify persistent or progressive impairments that suggest a chronic disease trajectory.^7^^,^^65^ Within this window, psychological screening tools mentioned previously can detect clinically significant symptoms^8^^,^^43^^,^^53^^,^^57^^,^^62^ (Fig. 2)*.* In tandem, risk stratification models can guide decisions regarding anticoagulant therapy continuation or modification, balancing bleeding risks against the threat of recurrent VTE.^15^^,^^66^, ^67^, ^68^

For patients with persistent dyspnoea, initial investigations must focus on ruling out CTEPH.^7^^,^^65^ The diagnostic approach typically begins with transthoracic echocardiography to evaluate right ventricular function and estimate pulmonary artery pressures.^7^^,^^10^^,^^65^ A V/Q scan can further delineate residual perfusion defects; if these findings sustain suspicion, right heart catheterisation coupled with detailed imaging (CTPA) provides definitive haemodynamic data and helps locate surgically accessible clots.^7^^,^^10^^,^^65^ In patients whose resting haemodynamics remain normal, yet who exhibit exercise-related symptoms, additional testing, such as CPET or stress echocardiography, can uncover subclinical pulmonary vascular limitations or subtle right ventricular dysfunction^7^^,^^44^, ^45^, ^46^, ^47^ (Fig. 1)*.*

The ICHOM-VTE standard sets four main outcome domains.^57^ Of these, the most relevant to managing individual patients are patient-reported outcomes (PRO), which capture quality of life, functional status, pain and dyspnoea severity, psychosocial wellbeing and treatment satisfaction. These metrics provide actionable insights for tailoring care to the patient’s specific needs. The Attend-PE model provides a real-world example of implementing the above.^34^ In this feasibility study, patients receive structured education and monitoring at key intervals (eg during the first few weeks, at 3 months, and as needed afterwards) to evaluate symptoms, adjust anticoagulant therapy, and address comorbidities or psychological concerns. Group-based education sessions also encourage relatives’ participation, aiming to bolster social support and aid communication between patients and healthcare professionals. Meanwhile, PRO questionnaires in Attend-PE highlight persistent needs, such as exercise intolerance, chronic dyspnoea or anxiety, and guide further referrals (to physiotherapy, general practice, or mental health services) when required. While Attend-PE has shown promise as a feasibility study, future research is needed to evaluate the long-term effectiveness of this intervention in improving clinical outcomes and patient quality of life.

**Fig. 3:**
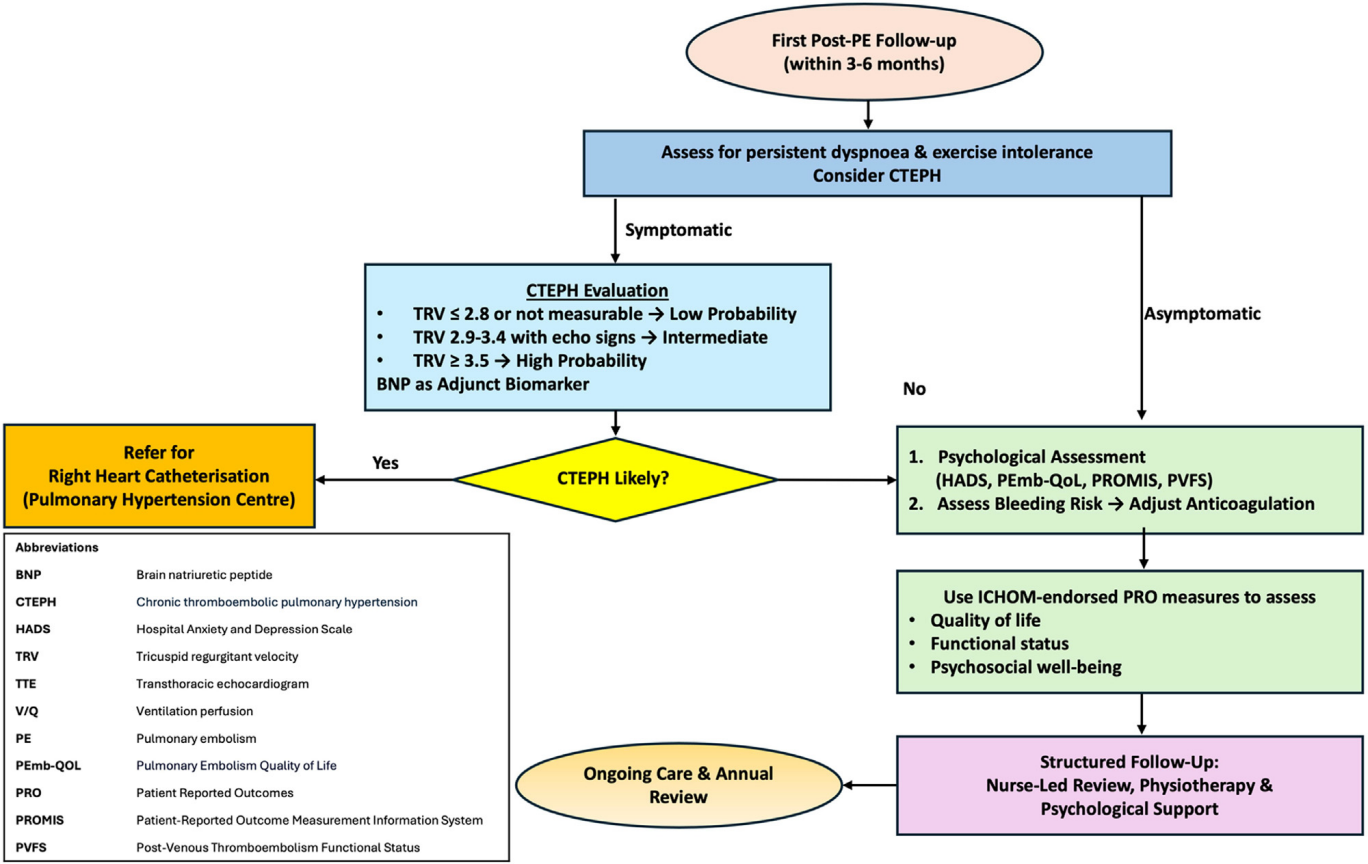
Proposed care model for post-PE management.

The standard of care, therefore, should entail systematic deployment of ICHOM-endorsed measures, augmented by the kind of structured, nurse-led follow-up demonstrated in Attend-PE. At each milestone (3, 6 or 12 months, and annually thereafter), clinicians incorporate PRO data alongside objective cardiopulmonary assessments to personalise treatment plans, guide anticoagulant intensity, and address both physical and mental health. Where indicated, dedicated rehabilitation programmes, akin to those supported by the Attend-PE model, help against deconditioning, reduce dyspnoea, and improve overall fitness.^28^^,^^30^^,^^35^ Concurrently, screening for or post-PE psychological sequalae fosters an environment where psychological challenges are proactively managed^26^^,^^57^^,^^62^ (Fig. 3)*.*

## Conclusion

Pulmonary embolism increasingly reveals itself as a condition with chronic implications. Recognising it as a chronic disorder necessitates a comprehensive, multidisciplinary approach that extends beyond acute management to encompass long-term strategies aimed at improving quality of life, reducing morbidity, and addressing psychological and functional impairments. By embracing this chronic care framework, clinicians can better navigate the complex trajectory of PE, ensuring holistic and sustained care that aligns with the evolving needs of patients.

## Funding

This article did not receive any specific grant from funding agencies in the public, commercial, or not-for-profit sectors.

## CRediT authorship contribution statement

**Gerard Gurumurthy:** Writing – original draft. **Lianna Reynolds:** Writing – original draft. **Kerstin de Wit:** Writing – review & editing, Conceptualization. **Lara N. Roberts:** Writing – review & editing. **Jecko Thachil:** Writing – review & editing, Conceptualization.

## Declaration of competing interest

LNR received speaker fees from Bayer, Chugai and Viatris.If there are other authors, they declare that they have no known competing financial interests or personal relationships that could have appeared to influence the work reported in this paper.
